# Use of Plaster-of-Paris in the Treatment of Club-Foot

**Published:** 1882-01-20

**Authors:** P. S. Connor

**Affiliations:** Chicago; Professor of Anatomy and Clinical Surgery, Medical College of Ohio


### USE OF PLASTER-OF-PARIS IN THE TREATMENT OF CLUB-FOOT.

BY P. S. CONNOR, M. D.,
**Professor of Anatomy and Clinical Surgery, Medical College of Ohio.**

That the deformity of club-foot may be corrected, it is necessary (1) that the distorted parts be put in normal position, and (2), that they be kept there; and, just in proportion as any plan of treatment accomplishes these indications, it is a proper one to be employed. Every one knows that in the great majority of congenital cases it is possible, during a variable but quite considerable time after birth, by manipulation, to bring the foot into its natural place; and, could it be sufficiently long and steadily held there by the hand of parent or nurse, rectification of the deformity would almost to a certainty result. What can be substituted for such gentle yet efficient hand-pressure? Ordinary bandages and adhesive plaster strips have been employed, and, in many cases, with advantage; but not infrequently they have been found inefficient, and adhesive strips often very much irritate the delicate skin of the foot and leg. As a rule, club-foot apparatus are not applied until the child is a few or several years old, when the deformity has become quite well developed, and when frequently strongly contracted tendons and more or less altered joint-surfaces much complicate the case. Against all the forms of these mechanical appliances several objections lie. In the first place, oftentimes they are badly adjusted, and in the majority of instances they do not maintain the correction made when they are applied; next, making pressure generally upon only a limited number of points, their use is very apt to be followed by local irritations, with resulting ulcers or callosities; again, they are expensive, and often require much repairing, and very many club-foot patients are the children of poor parents.

Recognizing the existence of these disadvantages, I have, for several years past, been experimenting with the plaster-of-Paris, recommended long ago by Little; the youngest subject upon which it has been applied being two weeks old. The success which has attended these experiments leads me to believe that this method of treatment may, in many cases at least, be advantageously employed. The application of it is easy; an ordinary plaster roller being turned over the foot, ankle, and lower half of the leg. In quite young patients a layer of cotton may with advantage be laid on, and the bandage put over it; in older ones the latter should be placed directly on the skin. The thickness of the covering must of course vary with the amount of deformity and existing muscular power. Before the plaster begins to set, the foot is to be brought as near its normal position as can be done without causing any decided pain, and held in place until the hardening is complete. Great care must be taken not to attempt too much at first. Preliminary tenotomy is to be made or not, according to circumstances, but is seldom required in young subjects. At each subsequent dressing more and more rectification is to be effected. Removal of the bandage will be required ordinarily in from three to six weeks, and the treatment, so far as I have been able to determine, must be continued for from six to eighteen months, and afterwards a half foot with stiffened sides, or the usual club-foot shoe worn, the former being generally sufficient to prevent any reproduction of the deformity.

Two objections have been made to this method of treatment: first, that the dressing is a heavy and clumsy one; and, second, that the proper development of the foot will be prevented. If properly applied, the weight of the plaster bandage is not so great as to prevent ready use of the limb if the patient is old enough to run about; and I have yet to see a single case in which the growth of the foot has been interfered with, or in which flattening of the arch has been produced. The advantages of the method are, that it is efficient, cheap, distributes the pressure which must be made in order that the foot shall be brought round and kept in place, can be employed at a very early period, can be put in practice by any one of ordinary skill and judgment, and does not necessitate calling in the aid of the surgical instrument maker, to whom oftentimes the case is turned over with instructions to "put on a club-foot shoe," as though it was to be treated simply with leather and steel. If it be thought advisable to have the foot daily bathed and rubbed, instead of the plaster roller, the "Bavarian" dressing might be applied; but I have never used it, preferring that the pressure should be continuous for as long a period as possible, with only such intermissions as are absolutely required for the reapplication of the bandage. When the deformity is of long standing, and large callosities are present from pressure, either of a previously-worn shoe, or of the weight of the body in standing and walking, special care must be taken in applying the dressing, and rectification must be very gradually brought about; otherwise ulceration will occur, and, as a consequence, the treatment may, and probably will, have to be entirely suspended until the ulcer heals, and, as generally happens under such circumstances, whatever the method pursued, the malposition will rapidly be reproduced.—[Medical News.

